# Supplementary material for: When shared concept cells support associations: Theory of overlapping memory engrams
Source: PLoS Comput Biol. 2021 Dec 30;17(12):e1009691. doi: 10.1371/journal.pcbi.1009691 (PMC8754331; doi:10.1371/journal.pcbi.1009691)
Supplement: S1 Text — (PDF) [file pcbi.1009691.s001.pdf]

# When shared concept cells support associations: theory of overlapping memory engrams


Chiara Gastaldi[1*], Tilo Schwalger[2 ‡], Emanuela De Falco[3], Rodrigo Quian Quiroga[4,5], Wulfram Gerstner[1‡]

**1** School of Computer and Communication Sciences and School of Life Sciences, École Polytechnique Fédérale de Lausanne, Lausanne, Switzerland
**2** Institut für Mathematik, Technische Universität Berlin, Berlin, Germany
**3** School of Life Sciences, École Polytechnique Fédérale de Lausanne, Lausanne, Switzerland
**4** Centre for Systems Neuroscience, University of Leicester, Leicester, UK
**5** Peng Cheng Laboratory, Shenzhen, China

‡These authors jointly supervised this work.
* chiara.gastaldi@epfl.ch


## Supporting information

Experimental studies measure the fraction of shared memory cells $c$. Such fraction of shared neurons can be interpreted as the probability that a neuron that already responds to one concept also responds to a second one. Formally, the fraction of shared neuron is the *conditional* probability $\mathrm{Prob}(x^2 = 1|x^1 = 1)$, where $x^\mu$ are binary variables that indicate whether a neuron belongs to the cell assembly representing concept mu ($\xi_i^\mu = 1$), or not ($\xi_i^\mu = 0$). In other words, $c$ is the fraction of shared neurons *relative* to the total number of neurons in the assembly under consideration. We define the overlap n between two cell assemblies as the absolute number of neurons that are shared between two cell assemblies $\mu$ and $\nu$: $n = \sum_{i=1}^{N} \xi_i^\mu \xi_i^\nu = c\gamma N$, where $\gamma$ is the fraction of neurons that take part in a given memory assembly. Note that $c\gamma$ is the *joint* probability that a neuron belongs to two memory assemblies: $P_{11} = \mathrm{Prob}(x^2 = 1, x^1 = 1)$. In the case of two *independent* random patterns $\vec{\xi}^\mu$, $\vec{\xi}^\nu$, the probability that a neuron belongs to both assembly $\mu$ and $\nu$ is $\gamma^2$. We refer to this scenario as the chance level. A chance level, the number of shared neurons (overlap) is $n = \gamma^2 N$ and, hence, the fraction of shared neurons is $c = \gamma$.

From a probabilistic point of view, the fraction of shared neurons c is uniquely determined by the Pearson's correlation coefficient $C = \frac{\mathrm{Cov}(\xi_i^1, \xi_i^2)}{\mathrm{Var}(\xi_i^\mu)}$. The fraction of shared neurons $c$ can be related to the Person's correlation coefficient $C$ uniquely: $c = n/(\gamma N) = \gamma + (1 - \gamma)C$. In the derivation of the derivation of the mean-field equations for overlapping memory engrams, we will refer to C simply as the correlation.

**Maximal fraction of shared neurons between memory engrams** In the Methods, we introduce the concept of the critical fraction of shared neurons as the fraction of shared neurons below which each pattern has a separated basin of attraction and above which the basins of attraction merge. The latter means that memories are indistinguishable. As mentioned in the introduction each fraction of shared neurons corresponds to a specific correlation value, so we can equivalently speak of critical correlation between memory patterns.

In the super critical regime $C > C_{\max}$, the activation of patter one triggers, with some delay, the activation of the correlated pattern 2, as shown in Fig 1D. The activation delay of pattern 2 can be quantified using the dynamical mean-field in Eq. (42). In panel A of S1 Fig we show the evolution of the system in the phase plane, before, during and after the stimulation respectively. In panel B of S1 Fig we can see the activation of $m^2$ due to the super-critical correlation with $m^1$ as predicted by the mean-field theory. We choose to quantify the time delay between the activation of pattern one and that of pattern two, by comparing the $m^1(t)$ and $m^2(t)$ lines in panel B of S1 Fig when they cross the value $m^1 = m^2 = \hat{h}_0$: time gap between the two crossing times defines the delay. Indeed, the dimensionless parameter $\hat{h}_0$ marks the point where the $m^2(t)$ curve becomes steeper, or, in the phase-planes, it is close to the ghost of the fixed point corresponding to the single retrieval state.

**Association chains** We estimate the range of correlations such that association chains are possible. That is, correlation should be large enough to trigger the activation of the next pattern, but not so large that the basin of attraction of single patterns merge. The strength of the global inhibition $J_0(t)$ varies slowly between its maximum and its minimum. Note that, when $J_0(t)$ is clamped at its minimum in panels B,D,F,H of S3 Fig left hand side, the double retrieving state is not present. The lack of the double retrieving state is a consequence of the introduction of the global inhibitory feedback, which leads to competition between the two assemblies and a winner-take-all response. The value of correlation, $C_{\max}$, that makes the two single retrieval states disappear can be read off from the bifurcation diagram in panel A of S2 Fig. It sets the upper bound of the useful correlation range and strongly depends on the value of mininal global inhibitory feedback, $\min(J_0)$. When we want to estimate the smallest value of correlation, $C_{\min}$ so that hopping between attractors is possible, we consider the situation when the global inhibition is clamped at its maximum and find the minimal correlation such that the system exhibits a *transition state*. The transition state is visible in panels D,H in the right hand side of S3 Fig, but it is not present in panel F of S3 Fig, since $C = 0$. Thus, the lower bound of correlation $C_{\min}$ is estimated by the left end of the stable diagonal branch of fixed points in the bifurcation diagram (panel B of S2 Fig).

In S3 Fig we compare the effect of different sparseness and correlations on the when hopping from one attractor to the next. For very sparse patterns, $\gamma = 0.002$, the transition is sharper (panel H of S3 Fig) and we observe the same dynamics (panel G of S3 Fig). While chains of associations are not possible for $C = 0$. Indeed, in panel B of S3 Fig we assume $\vec{\xi}^1$ and $\vec{\xi}^2$ to be independent ($C = 0$) and the state is never able to leave the basin of attraction of pattern 1. The reason is clear from the phase-planes in panel A of S3 Fig, when $J_0$ is at its maximum, $m^1$ is decreased in value until a stable fixed point, where still $m^2 = 0$: the second pattern is not even partially activated, and when the inhibition decreases, the system state falls back into the first pattern basin of attraction. At the contrary, when enough correlation is added, any activation of $\xi^1$ implies a partial activation of $\xi^2$. When $J_0$ reaches an high value, the system is pushed in a neutral state where both $m^1 = m^2 \sim C$. At the subsequent decrease of $J_0$, the system might fall in either of the two single retrieval states, but adaptation breaks the symmetry and pushes the system towards the pattern that was not activated yet.

In S4 Fig we compare full network simulation with dynamical mean-field for $p = 2$ and $p = 4$. The mean-field and the full simulation match.

**Parameters choice** We have discussed that the critical correlation between patterns depends on two dimensionless parameters: the rescaled threshold $\hat{h}_0 = h_0/(Ar_{\max})$ and the rescaled steepness $\hat{b} = Ar_{\max}b$. While these parameters have so far not being

estimated for human Hippocampus, the transfer function has been fully characterized for pyramidal neurons of the macaque's IT cortex [1]. We therefore computed the critical correlation for this physiological parameter set. First, in [1] the input is processed by the combination of two sigmoids. We have chosen the parameters of our single sigmoidal transfer function such that it fits the combination of sigmoids used in [1]. Moreover, whereas in [1] patterns with $N(0,1)$ Gaussian distributed elements have been considered, in our theory we assumed binary patterns. To match roughly match the two settings, we have chosen $\gamma = N(0,1)(h_0)$, where $h_0$ is estimated from the fitted transfer function. We obtained $\gamma = 0.0375$. In panel A of S5 Fig we show the phase-plane for $C = 0$ (or equivalently $c = \gamma$) and in panel B of S5 Fig we provide the bifurcation diagram from which the critical correlation is extracted (equivalent to that in Fig 9B).

# References


1. Ulises Pereira and Nicolas Brunel. Attractor dynamics in networks with learning rules inferred from in vivo data. *Neuron*, 99(1):227 – 238.e4, 2018.
