# Supplementary figures and images for: When shared concept cells support associations: Theory of overlapping memory engrams
Source: PLoS Comput Biol. 2021 Dec 30;17(12):e1009691. doi: 10.1371/journal.pcbi.1009691 (PMC8754331; doi:10.1371/journal.pcbi.1009691)

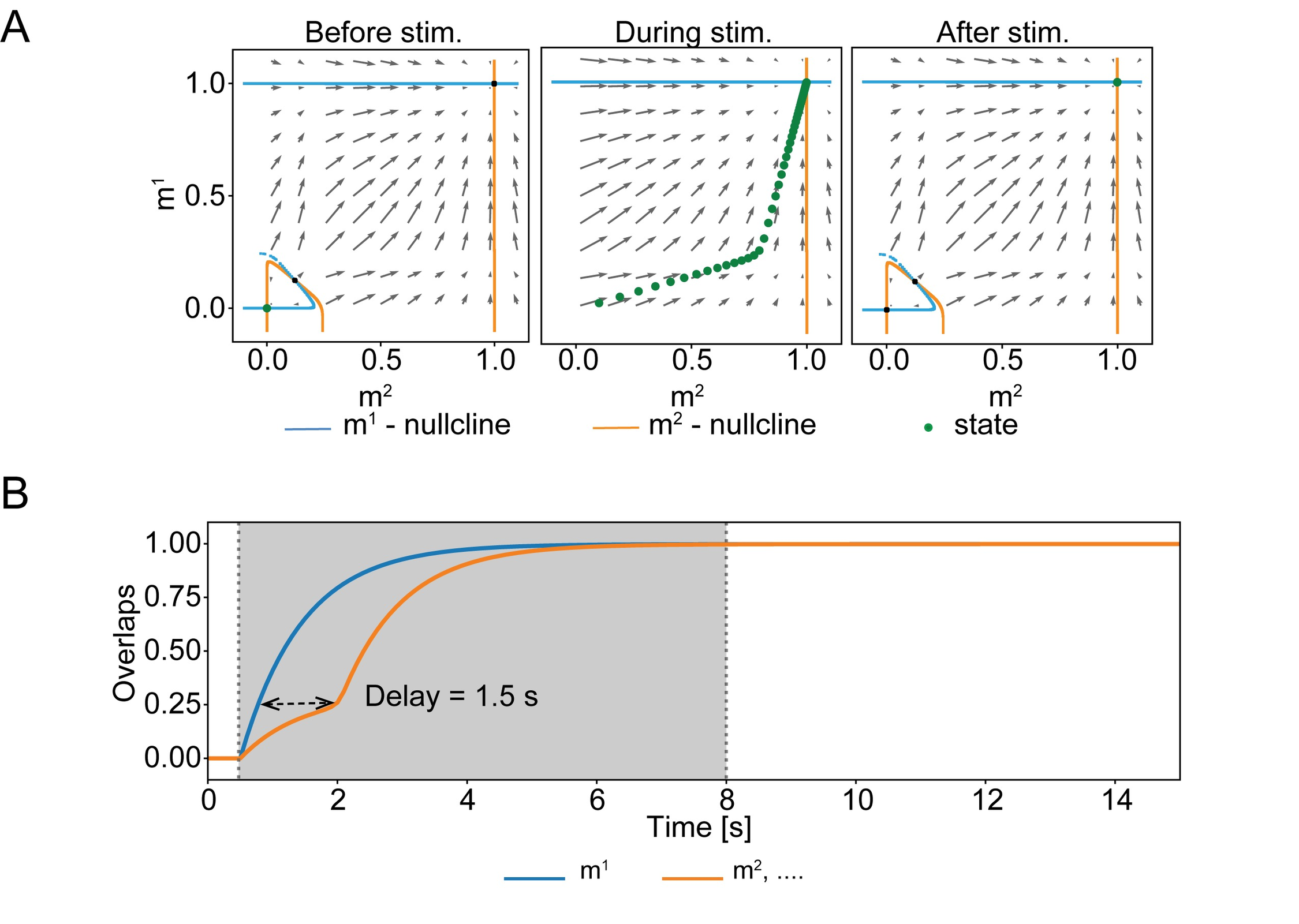

Supplement: S1 Fig — The system is initialized in the rest state. During the stimulation period (0.5–8s) m1(t) receives external input. A) The system state is plotted in the phase-plane before, during and after stimulation respectively. B) The delay between the activation of m1(t) and m2(t) is highlighted. Parameters: γ = 0.002, b^=100, h^0=0.25, rmax = 1, τ^=τ=1rmax, α = 0, C = 0.2. (TIF) [file pcbi.1009691.s002.tif]

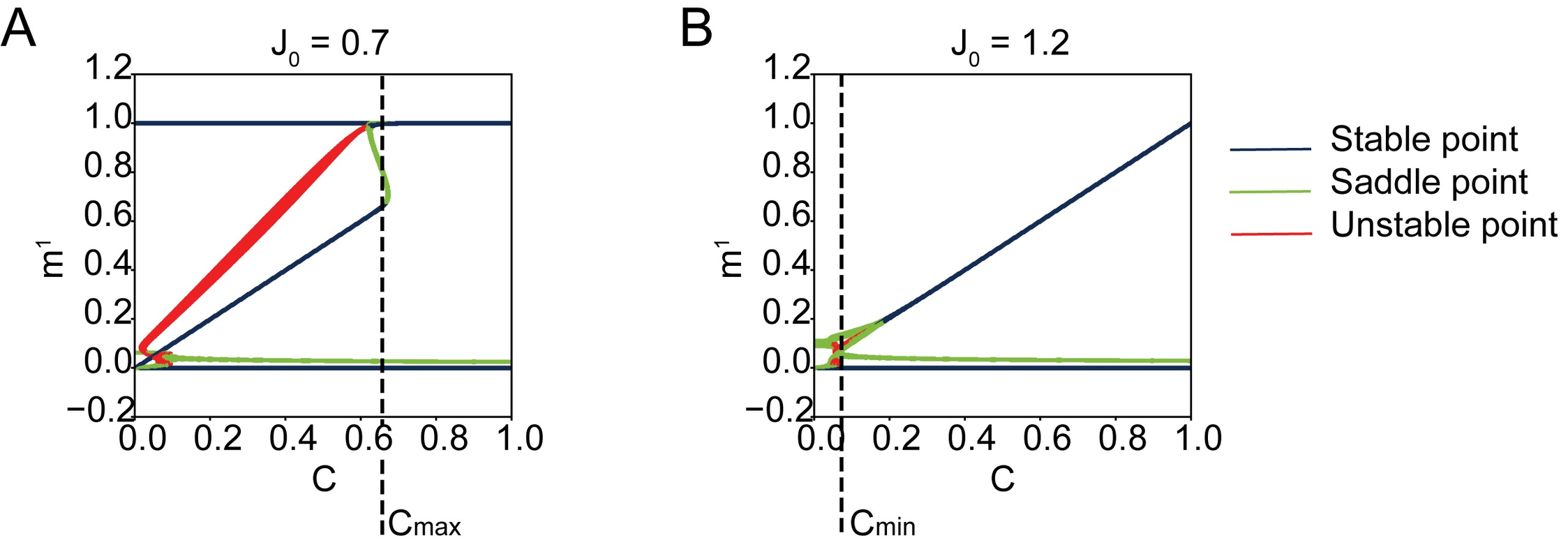

Supplement: S2 Fig — A) Estimation of the maximum correlation, which correspond to the loss of the two single retrieval states, when J0 is lowest. B) Estimation of the minimum correlation, which corresponds to the creation of the stable fixed point at m1 = m2 > 0, when the inhibition J0 is at its maximum. In both A and B adaptation is frozen and θ = 0. Parameters: γ = 0.002, α = 0, b^=50, h^0=0, min(J^0)=0.7, min(J^0)=1.2. (TIF) [file pcbi.1009691.s003.tif]

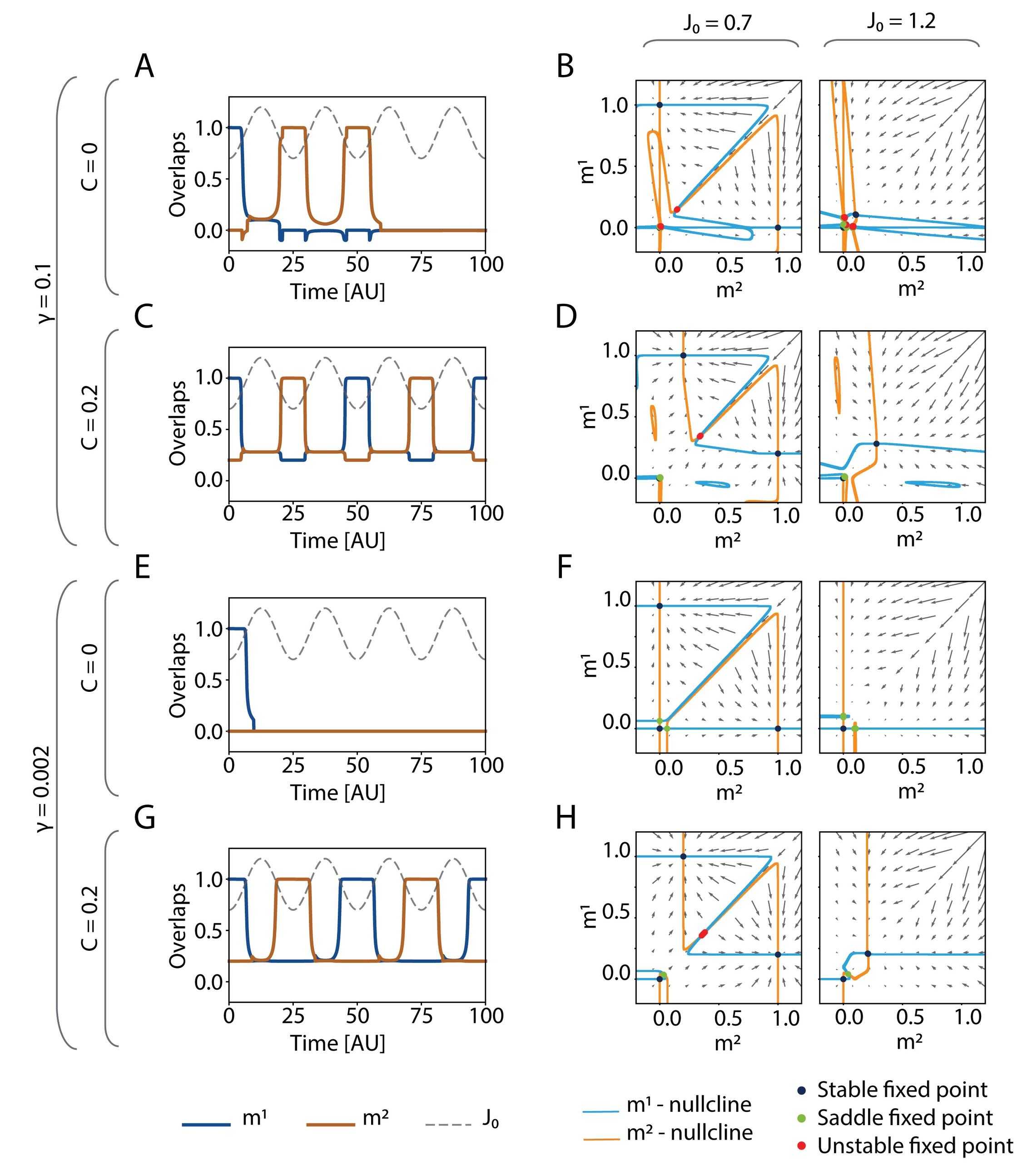

Supplement: S3 Fig — A) Dynamical mean-field solutions for m1 and m2 in the case of two independent patterns. B) Phase planes corresponding to the minimum (J0 = 0.7) and maximum (J0 = 1.2) value of inhibition in the case of two independent patterns. C,D) Same as A and B, but for correlated patterns C = 0.2. Parameters in A—D: γ = 0.1, α = 0, b = 100. E, F) Same as C and D but in the low activity regime and for independent patterns. G, H) Same as C and D but in the low activity regime. Parameters in E—H: γ = 0.002, α = 0, b = 100, τθ = 45, T = 0.015, TJ0=25. For the dynamics: resolution = 200, factor = 1. For the phase-planes: resolution = 1000, factor = 1, upper bound = 1.2, lower bound = -0.2 (same as Figs 2 and 4). (TIF) [file pcbi.1009691.s004.tif]

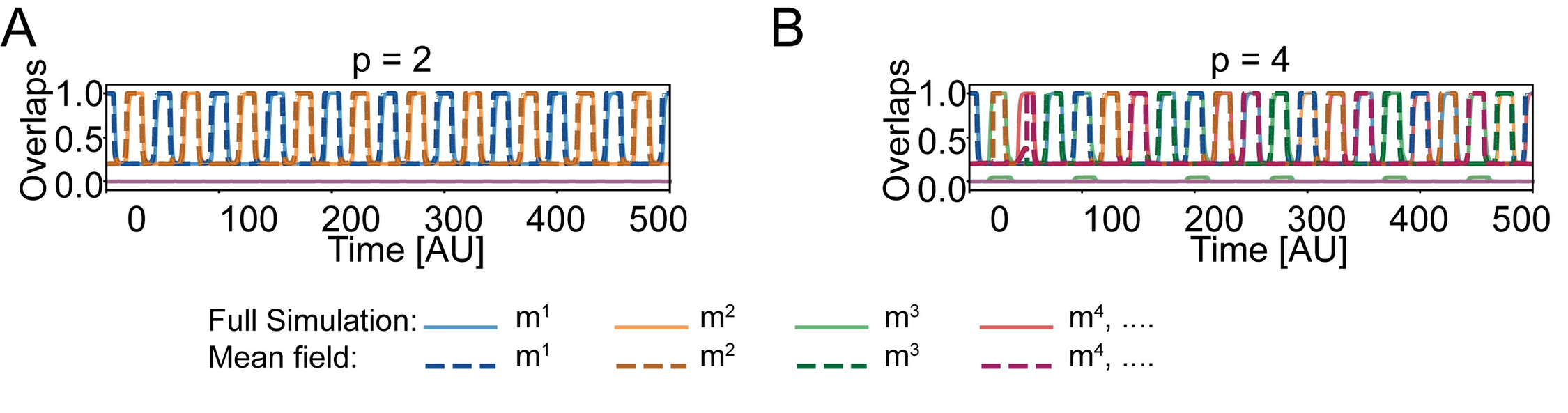

Supplement: S4 Fig — Retrieval dynamics in the presence of adaptation according to the mean-field equations (dashed lines), and comparison with Fig 4A (shaded solid lines) A) Only two patterns are correlated. B) Four patterns are correlated. Parameters: N = 104, P = 16 in full network simulations and α = 0 in mean-field. γ = 0.002, τθ = 45, T = 0.015, TJ0=25 in both. (TIF) [file pcbi.1009691.s005.tif]

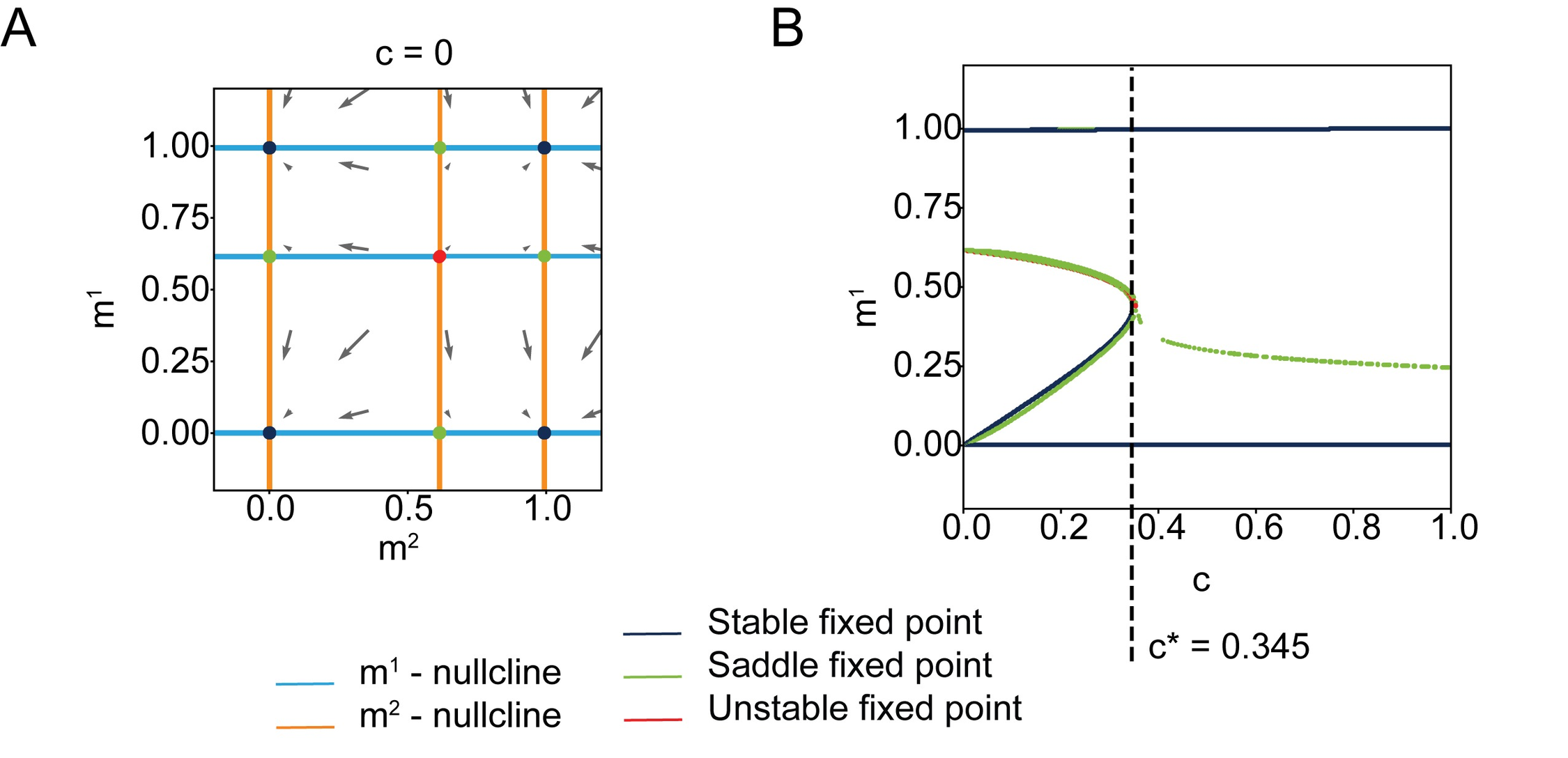

Supplement: S5 Fig — In A and B the transfer function parameters are taken as those of function ϕ in [24]: A = 3.55, rmax = 76.2, b = 0.82, h0 = 2.46. On the other hand, in C and D I estimated the parameters of a Sigmoid function that fits the function f(ϕ) in [24] as follows: A = 3.55, rmax = 0.83, b = 4.35, h0 = 1.7. In all plots γ = 0.001. A and C) The phase-plane for c = 0 shows the position of fixed points. B and D) Bifurcation diagram and critical fraction of shared neurons according to different parameter choices. (TIF) [file pcbi.1009691.s006.tif]

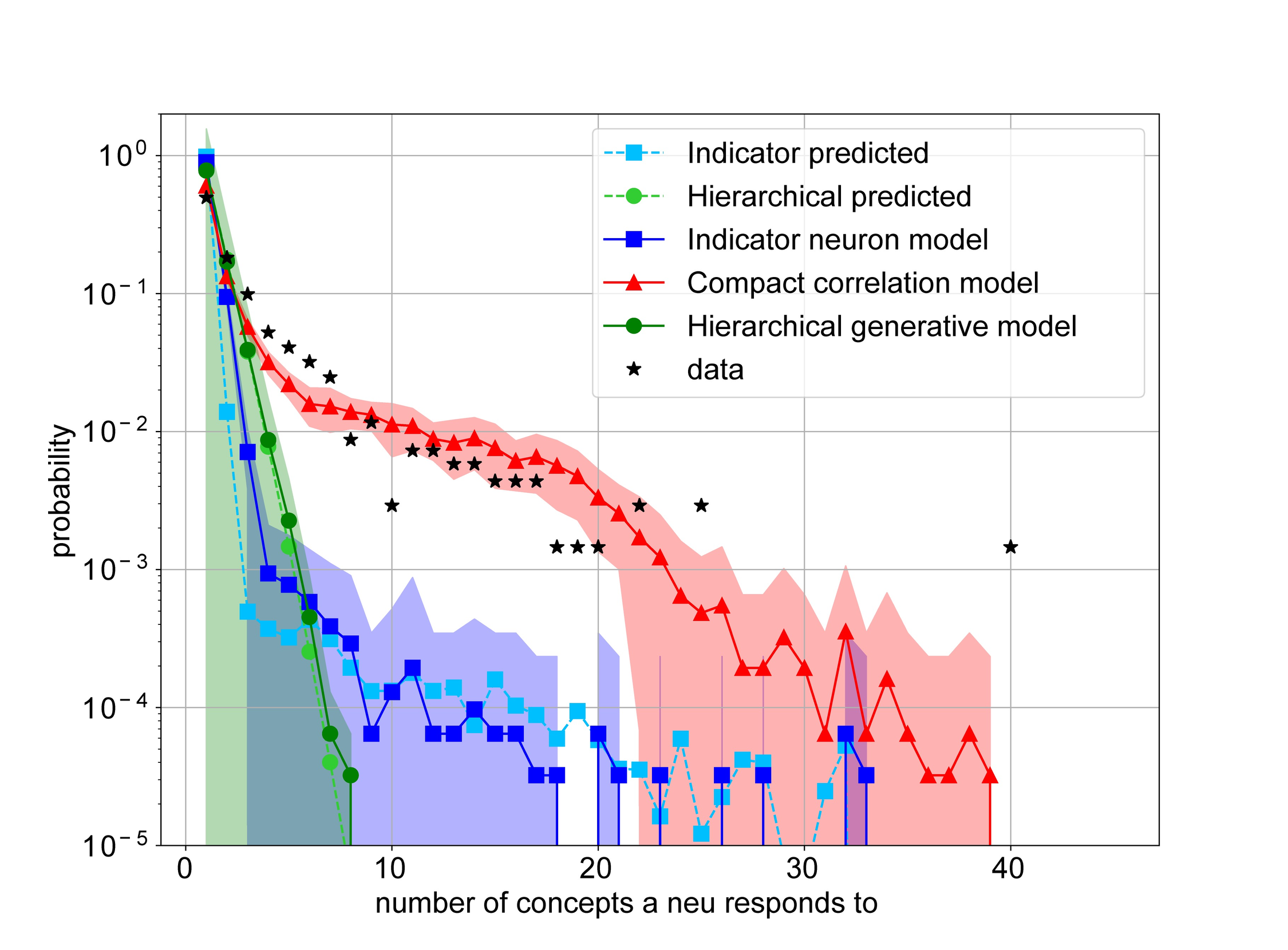

Supplement: S6 Fig — Probability of finding a neuron responding to a given number of concepts as measured from experimental data (black stars), predicted by the three algorithms (as in Fig 6, the area between error bar of one standard deviation is shaded) and theoretically forecast for the indicator neuron model (light blue) and for the hierarchical generative model (light green) obtained from Eq (99). The theoretical predictions are not smooth curves due to choice of matching the subgroups sizes to the dataset. (TIF) [file pcbi.1009691.s007.tif]
