# Supplementary material for: When shared concept cells support associations: Theory of overlapping memory engrams
Source: PLoS Comput Biol. 2021 Dec 30;17(12):e1009691. doi: 10.1371/journal.pcbi.1009691 (PMC8754331; doi:10.1371/journal.pcbi.1009691)
Supplement: Attachment — Submitted filename: Answer to Reviewers.pdf [file pcbi.1009691.s008.pdf]

Answer to Reviewer 1:

R1.1. Section - association chains

In the new version of the paper we write: *The number of concepts in the cycle depends on the time scale of adaptation: in Fig. 4 we use* $\tau_\vartheta \sim 2T_{J_0}$*, which determines a minimum cycle size of 3 concepts. In previous studies [9–12,26], each memory engram involved a large fraction (γ = 10%) of neurons so that transitions could rely on the number of units shared by chance. The overlaps between memory assemblies may vary due to finite size effects. The concept that shares the biggest overlap with the active one, is activated next, until the association chain falls into periodic cycle of patterns*.

You can find this paragraph at lines 209-220 of the new submission (clean file), or lines 211-225 of the file with tracked changes.

In previous studies ([9–12, 26], in the paper) the overlaps between memory assemblies varies due to finite size effects. The concept that shares the biggest overlap with the active one, is activated next, until the association chain falls into a cycle.

On the other hand, in the mean field limit, all associated concepts share exactly the same number of neurons and the order of the reactivation is random and only depends on the initial condition, Fig 4B.

In both cases, the number of concepts in the cycle depends on the time scale of adaptation: in Fig. 4 we use $\tau_\theta \sim 2T_{J_0}$, which determines a minimum cycle of 3 concepts.

R1.2. Section - How does a network embed groups of overlapping memories?

We have expanded the section between lines 290 and 306 of the new submission (same lines of the file with tracked changes) with a brief intuition for each algorithm and an explanation for the main difference between the two hierarchical models considered.

In the new version of the paper we write: *First we consider an iterative overlap-generating model a non-hierarchical model, in which we impose a fixed target number of shared concept cells as the only condition. Second, we consider two hierarchical models, in which every subgroup of associated memory patterns is derived by a single "parent" pattern, which does not take part in the subgroup. In the hierarchical generative model, only neurons that belong to the parent pattern can be contribute to the neural representation of the patterns in the subgroup. On the contrary, in the second indicator neuron model, the parent pattern is composed of indicator neurons that have a fixed probability* λ_{ind} *of appearing in each of the subgroup's patterns. Non-indicator neurons can also take part in the representation of the subgroup's patterns with a different probability Ω. In other words, in the hierarchical generative model, neurons that do not belong to the parent pattern are excluded from the representation of all of the associated patterns in a subgroup, while in the indicator neuron model, no neuron is excluded from contributing to the representation of the subgroup.*

R1.3 Section - Robustness to heterogeneity

In lines 346-353 (both files) of the new version of the paper we write: *We have explored lower values of connection probability d, to approach a more bio-plausible regime [27]. However, increasing the dilution of the connections takes the dull network simulations away from the mean-field regime in which the theory is valid. The problem could be overcame by increasing proportionally the size of the network size N, but the computational cost grows with the square of network size N. The optimization of the simulation of a very diluted attractor neural network is beyond the goals of the current work.*

We claimed that the model is particularly suitable to describe area CA3 of the hippocampus, which is known to be a very recurrent network. In fact, each neuron in this area is estimated to receive inputs from about 30'000 other neurons (Andersen et al., 2006). However, it is also estimated that there are about 2.8 million neurons in CA3, which means that a bio-plausible dilution would be of about $d = 0.01$, implying to drop of about 99% of connections starting from a fully connected scenario. The connectivity in the CA3 has been measured also in (Guzman et al., Science, 2016. DOI: 10.1126/science.aaf1836), where they estimate $d = 0.0092$.

Implementing such a diluted network posed a theoretical and a computational issue. First, since the highest we set the dilution, the more full network simulations with finite number of neurons are away from the mean-field regime in which the theory is valid. This is particularly true because the weight dilution adds up to the high sparseness of the memory patterns. Second, in order to reproduce such a regime in full network simulations, we would need to scale up the system and increase the number of neurons in the network. However, in trying to do so, we have encountered a computational problem: the running memory usage of full network simulation scales with the square of the number of neurons. The current version of the code (written in Julialang) uses about 16 GB of RAM for a full network simulation counting $N =$ 50'000 neurons, a million neurons network would require about 1620 GB of RAM which is of course not feasible. One option we have tested was to use Julialang's tool for sparse matrix computations. Such a tool decreases dramatically the request of memory, for the simulation of $N =$ 50'000 less than 3 GB are needed, but the price to pay is an increase of the computation time, which scales roughly with the square of the number of simulated neurons. Also, in this case the computational time needed to simulate a million neurons is prohibited. The optimization of the simulation of a very diluted attractor neural network is beyond the goals of the current work, but it would be an interesting future development.

Answer to Reviewer 2:

R2.1 - any pair has above chance correlations
In lines 416-427 of the new version of the paper we write the following:
We discuss the possibility of allowing all patterns to share the same amount of correlation at the very end of section "Overlapping background patterns", in the Methods. In this case, we show that the standard deviation of the background noise is proportional to P^2/N. If we make the standard mean-field assumption that both P and N tend to infinity with constant ratio $P/N = \alpha$, then the quenched noise due to the presence of background patterns would diverge.

However, we can define the memory load $\alpha' = P^2/N$ and we can assume that P and N tend to infinity, keeping α' constant. In this scenario, the network capacity is drastically reduced. Alternative approaches to take into account the fact that some neurons are more easily recruited, can be

1. To assign heterogeneous gain functions, therefore making some neurons more excitable,

2. To consider those neurons to be the "multi-responsive neurons" that we describe in section "How does a network embed groups of overlapping memories".

R2.2 – scaling laws of the number of retrieved patterns

It would be hard to reproduce the prediction of the Romani-Tsodyks model that the number of retrieved items scales sub-linearly with the number of presented item in our model. In fact, they base their calculation on a symmetrical similarity matrix and on the assumption that once an item is retrieved twice a retrieving cycle begins and this determines the total number of retrieved items. However, in our mean-field theory, we would have a block diagonal similarity matrix, which does not justify the assumption that the association chain will necessary end in a cycle (even though that is what we have observed in our simulations).

R2.3 – context-dependent disentanglement of memories

Thank you for the useful recommendation, we have added a reference to (Podlaski, Agnes & Vogels, bioRxiv 2020) at lines 391-395, where we write

In our framework the activation of one context favors the recall of concepts associated to it, and it can be qualitative compared to neuron-specific gating model proposed in [30], where the activation of one context defines a subset of available neurons.

General:

- All typos have been corrected.
- The link to the code and data has been inserted in the section "Numerical solutions".
